# Patients with episodic migraine without aura have an increased rate of delayed discounting

**DOI:** 10.1002/brb3.3367

**Published:** 2024-01-02

**Authors:** Lu Wang, Chenyang Dai, Manman Gao, Zhi Geng, Panpan Hu, Xingqi Wu, Kai Wang

**Affiliations:** ^1^ Department of Neurology The First Affiliated Hospital of Anhui Medical University Hefei China; ^2^ School of Mental Health and Psychological Sciences Anhui Medical University Hefei China; ^3^ Institute of Artificial Intelligence Hefei Comprehensive National Science Center Hefei China; ^4^ Anhui Province Key Laboratory of Cognition and Neuropsychiatric Disorders Hefei China; ^5^ Collaborative Innovation Center of Neuropsychiatric Disorders and Mental Health Hefei China; ^6^ Anhui Provincial Institute of Translational Medicine Anhui Medical University Hefei China

**Keywords:** delay discounting task, impulsivity, temporal discounting, triptans, ventral striatum, ventromedial prefrontal cortex

## Abstract

**Objective:**

This study aimed to explore decision‐making impulsivity and its neural mechanisms in patients with episodic migraine without aura (EMoA).

**Background:**

Previous evidence indicates increased impulsivity and altered reward processing in patients with chronic migraine and medication overuse; however, whether the same holds true for those with EMoA is unclear.

**Methods:**

Patients newly diagnosed with EMoA (*n* = 51) and healthy controls (HC, *n* = 45) were recruited. All participants completed delay discounting task, cognitive assessments, a questionnaire for headache profile, and resting‐state function magnetic resonance imaging scans. Resting‐state functional connectivity (RSFC) between the regions of interest and the entire brain was explored.

**Results:**

Patients with EMoA showed a steeper subjective discount rate than HCs (*F* = 4.74, *p* = .032), which was positively related to a history of migraines (*r* = .742, *p* < .001). RSFC among the ventral striatum (vSTR), ventromedial prefrontal cortex, and occipital cortex was lower in patients with EMoA than in control groups, which was correlated with history (*r*′ = .294, *p* = .036) and subjective discount rate (*r*′ = .380, *p* = .006). Additionally, discounting rates and RSFC between the vSTR and occipital regions were significantly abnormal in the triptan group than the non‐triptan group. Mediating effect analysis indicated a significant mediating effect in the change in RSFC between the vSTR and occipital status, history of triptan use, and subjective discount rate.

**Conclusion:**

This study further elucidated that an increase in delayed discounting rate exists in patients with EMoA and is related to the abnormality of the value processing network.

## Graphical abstract

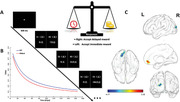

## 1 BACKGROUND

Migraine is a chronic neurological disorder characterized by moderate or severe headache attacks and reversible neurological and systemic symptoms (Dodick, 2018). According to a World Health Organization survey, migraine is most prevalent between the ages of 25 and 55 years, which is the third most prevalent disorder and second most specific cause of disability worldwide (Forouzanfar et al., 2016).

In addition to unilateral, throbbing pain, many different non‐headache symptoms occur during or between migraine attacks (Headache Classification Committee of the International Headache Society [IHS],^2018^ ; Tu et al., 2022). Several studies have examined the association between migraine and cognitive function (Chen & Wang, 2018; Karsan & Goadsby, 2021). Many have found a significant drop in cognitive efficiency in migraineur (Attridge et al., 2017; Batenhorst, 2001; Cady & Farmer, 2013; Ferreira et al., 2018; Pellegrino Baena et al., 2017; Reeves, 2000) However, most of these studies focused on chronic migraine, drug abuse migraine, and all phases of episodic migraine with aura from the premonitory through the recovery (post‐drome) phase, rather than on episodic migraine in patients without aura during intermission (Martins et al., 2012). Additionally, other features remain poorly explored in episodic migraine without aura (EMoA), including cognition and alterations in reward processing in the brain (Karsan & Goadsby, 2021), both of which, similarly to medication overuse headache and chronic migraine, could constitute risk factors for progression and relapse (Niddam et al., 2023).

Migraine sufferers seem to be impatient and impulsive, especially those with headaches and chronic migraine (Zhang et al., 2012). Impulsivity is an individual's strong emotional expression or lack of rational consideration of the result of an action (Potvin et al., 2015), which has an important influence on decisions (Wittmann & Paulus, 2008). The effects of increased impulsivity in decision making are often unpredictable and associated with mental disorders, economic crimes, drug abuse, and traffic accidents. For migraine sufferers, impatience may be closely associated with substance abuse, making them more likely to develop chronic migraines or substance abuse. Generally, impulsivity can be characterized as a disturbance in the balance between immediate reward and delayed gratification processes (McClure et al., 2004), which can be quantified by delay discount rates (Lempert et al., 2019). Delay discounting refers to the extent to which the value of a reward decreases as the delay to receipt increases. Steeper delay discounting rates are associated with numerous health conditions, such as medication overuse headaches, neurodegenerative diseases, addictive disorders, and obesity (Amlung et al., 2019; Elton et al., 2019; Garzon et al., 2023; Koban et al., 2023; Niddam et al., 2023; Wang et al., 2020; Yeh et al., 2021; Zeng et al., 2022). Patients with medication overuse headaches showed higher impulsivity (Niddam et al., 2023) and were associated with substance abuse, making them more likely to develop chronic or substance‐abuse migraines. However, whether there is an increase in delayed discounting during the intermission of attacks in patients with EMoA is unclear.

Delay discounting task (DDT), which includes reward valuation and impulsivity, is a common method for measuring delay discounting. It refers to presenting subjects with binary choices between a smaller reward given immediately and a larger reward given in the future and fits a model that predicts responses based on a hypothetical discount rate. The hyperbolic model is then adopted to quantify the delay discount rates, which fits data better than most other models do, even across species, and is the most used discount function in psychology and psychiatry (Zeng et al., 2022). In this model, *k* is a free parameter representing the discount rate and provides quantifiable metric for examining a complex phenomenon in which the subjective value decreases with the time delay. Generally, larger *k* values indicate a stronger preference for smaller or sooner rewards, whereas smaller *k* values indicate a less steep discounting of delayed rewards. Hence, DDT may be an effective method for quantitatively evaluating impulsivity in patients with EMoA. Additionally, the potential neuromechanisms of impulsivity in patients with EMoA should be explored.

Previous studies have linked impulsive behavior to the ventral striatum (vSTR), a critical component of the basal ganglia and involved in the mental processes associated with impulsivity. Considerable evidence suggests that the vSTR, a central node in the reward system, is predominantly involved in motivation/reward‐related processes, such as delayed reward valuation, summed value encoding, and immediate option preference processing (Wang et al., 2020). The vSTR activity response to positive and negative feedback as well as the functional connectivity intensity with other brain regions can predict an individual's impulsive choice (Knorr et al., 2020; Lee et al., 2023). The vSTR helps regulate the activity and behavioral effects of dopaminergic and serotonin neurons by receiving and filtering excitatory inputs specific to multiple brain regions (Christoffel et al., 2021). Thus, the vSTR plays a central role in impulsive decision‐making. Resting state functional magnetic resonance imaging (rs‐fMRI) provides a method to understand neural mechanisms in vivo. Therefore, we also collected rs‐fMRI images of patients with migraine to explore changes in brain activity and functional connectivity related to impulsive decision‐making in patients with EMoA.

Previous research focused more on chronic migraine or migraine with aura than on EMoA, particularly regarding social cognitive function (Bottiroli et al., 2023; Estave et al., 2021; Gu et al., 2022). This study was designed to explore decision‐making impulsivity and its neural mechanisms in EMoA patients to reveal more clinical features and clarify whether increased delay discounting is also present in EMoA patients. Further, this study aimed to explore different medications’ effects on decision‐making in patients with EMoA.

## 2 MATERIALS AND METHODS

### 2.1 Overview

This descriptive study was conducted by interviewing patients treated at a Neurology Clinic from the First Affiliated Hospital of Anhui Medical University, Hefei. The main study included patients with EMoA and healthy controls (HCs) who underwent task‐based and multi‐model magnetic resonance imaging (MRI). To reduce the effects cyclical hormones, all female participants were instructed to undergo the test before ovulation (the time of ovulation was regarded as 14 days before menstruation). This study was approved by the Ethics Committee of the Anhui Medical University.

### 2.2 Participants

Inclusion criteria were (1) age between 20 and 50; (2) score of mini‐mental state estimate (MMSE) ≥24; (3) patients diagnosed according to International Headache Society (third) criteria (IHS, 2018); (4) history of migraine >0.5 year; (5) acute medications were limited to nonsteroidal anti‐inflammatories, triptans or unused drugs; and (6) provided written informed consent to participate.

Exclusion criteria were (1) illiteracy and age>50 years, (2) diagnosis of dementia or MMSE score <24, (3) organic brain dysfunction or psychiatric disorder (i.e., neurological diseases that influence cognitive function, head trauma, schizophrenia, depression, and anxiety), (4) patients with other chronic pain (i.e., sciatica, rheumatoid arthritis), and (5) alcohol abuse or dependence.

Each patient was interviewed personally for the evaluation of demographic and clinical data and neuropsychological testing for cognitive evaluation. Individuals matched for sex, age, and education, with no diagnosis of migraine, and whose parents and siblings were not diagnosed with migraine and met the exclusion criteria were used as controls.

### 2.3 Clinical data

#### 2.3.1 General characteristics

Data on age, sex, education, and family history were obtained during interviews. The patients were also assessed for comorbidities such as depressive and anxiety disorders (Diagnostic and Statistical Manual of Mental Disorders—DSM‐5), alcohol abuse (defined as moderate drinking [<20 g/day] and risky drinking [20–60 g/day]), current smoking status (no [less than one cigarette puff per day]/frequent [more than one cigarette puff per day]) and family history (parents or siblings diagnosed with migraine), and the impact of these conditions on their lives.

#### 2.3.2 Headache assessment

Patients answered a series of questions regarding their experiences of headaches, including diagnosis of migraine, frequency and intensity of headache attacks, doses of daily medications, and analgesic use. We used the McGill pain questionnaire (SF‐MPQ), which included the pain rating index (PRI) sensory index (PRI‐S), PRI affective index (PRI‐A), and the visual analog scale (VAS) to assess pain in patients with EMoA. Participants reported headache severity of their current and mean headache pain using a 100‐mm VAS anchored with labels “no pain at all” on the left and “worst imaginable pain” on the right. The HIT‐6 (Headache Impact Test) was included to assess the effect of participants’ headaches on their daily lives.

#### 2.3.3 Neuropsychological assessment

Patients and controls were subjected to standardized neuropsychological assessments, including global cognition (MMSE), attention function (Digital Span Test‐Forward/Backward, DST‐F/B), executive function (Stroop Color Word Interference Test‐pot/word/color word, SCWT‐pot/word/color word), and semantic memory (Verbal Fluency Test‐animal/vegetables, VFT‐A/V). The Hamilton Anxiety Rating Scale (HAMA) and Hamilton Depression Rating Scale were used to assess participants’ possible anxiety and depressive symptoms.

### 2.4 Delay discounting task (DDT)

Participants were administered a computerized DDT. The trial started with a warning cue and a fixation point displayed on a screen for 800 ms, followed by an offer of an implicit choice between a smaller‐but‐sooner (SS) reward and a larger‐butlater (LL). Participants were instructed to choose between two alternatives. The SS meant a small reward that could be obtained immediately, whereas LL meant a larger reward later. The *k* values were used to assess temporal discounting and impulsive decision‐making behaviors (Lempert et al., 2019). Because the subjective discount factor k was non‐normally distributed, nonparametric tests or a log10 transform were used. A larger *k*‐value/lg‐*k* value corresponds to a steeper discount rate and indicates an increased impatience or impulsivity. More details are provided in the Supporting Information section.

### 2.5 Imaging data acquisition and analysis

Structural and resting state MRI images were acquired using a General Electric HD 750 W 3.0 T MRI scanner with an 8‐channel head‐coil (General Electric). Seed‐based resting‐state functional connectivity (RSFC) analysis was employed to explore neuromechanisms underlying choice impulsivity in migraineurs. Seed points were defined as spheres of 4‐mm radii and included the left vSTR (left vSTR MNI coordinates: −10, 10, 2) and right vSTR (10, 12, −4) (Niddam et al., 2023). rs‐fMRI analysis was performed using the advanced edition of the Data Processing Assistant for Resting‐State Functional software, based on the statistical parametric mapping software package (SPM12; www.fil.ion.ucl.ac.uk/spm). More details are provided in the Supporting Information section.

### 2.6 Statistical analysis

Data were analyzed using SPSS software version 26. The chi‐square or Fisher's exact test was used to analyze categorical variables according to expected frequency in cells. The Kolmogorov–Smirnov test was applied to analyze the numerical variables to define the type of variable distribution. Normally distributed data were analyzed using Student's *t*‐test or analysis of variance, and non‐normally distributed data were analyzed using the Mann–Whitney test. Analysis of covariance was adopted to compare subjective value discounts between the two groups. Mediating effects and Spearman's correlation analyses were also performed. Statistical significance was set at *p* < .05 (two‐tailed).

## 3 RESULTS

### 3.1 General characteristics

#### 3.1.1 Demographics

A total of 104 individuals were preselected and 8 of them excluded. Of these, four were selection failures due to the head‐motion (three in HCs, one in EMoA), two did not complete the DDT and two withdrew their consent to participate in the study. Finally, 96 participants included 51 patients with EMoA and 45 HCs. The demographic and clinical characteristics of the two groups are shown in Table 1. There were no differences in age, education, sex, or alcohol and smoking habits.

**TABLE 1:** Demographics, neuropsychological assessment, and delay discounting task (DDT) performance of episodic migraine without aura (EMoA) and healthy control (HC).

	HC	EMoA	*F/t*/*Z*/*χ* ^2^	*p*
Sex (F/M)	33/12	38/13	0.02^a^	.896
Age (years)	31.02 ± 9.32	31.31 ± 10.03	0.00^b^	.997
Education (years)	12.96 ± 3.78	12.31 ± 3.35	−1.22^b^	.223
Alcohol (frequent/no)	6/39	2/49	2.77^a^	.141
Smoking (frequent/no)	3/42	3/48	0.03^a^	.874
Family historical (yes/no)	Na	28/23	Na	Na
Total time of migraine pain (years)	Na	7.61 ± 6.86	Na	Na
Frequency Acute pain (days/month)	Na	4.63 ± 2.82	Na	Na
Headache frequency (per month)	Na	4.82 ± 2.96	Na	Na
Duration of headache (hours)	Na	25.33 ± 12.56	Na	Na
McGill pain questionnaire	Na	11.02 ± 5.20	Na	Na
Pain rating sensory index	Na	6.62 ± 3.08	Na	Na
Pain rating index affective index	Na	4.39 ± 2.89	Na	Na
Mean headache severity (0–10)	Na	5.82 ± 1.61	Na	Na
Headache intensity on scan day (0–10)	Na	2.52 ± 0.96	Na	Na
Headache impact test	Na	59.49 ± 8.33	Na	Na
Lg (*k*)	−2.37 ± 0.99	−1.92 ± 0.88	4.74^d^	***.032***
Mini‐mental state examination	29.89 ± 0.49	29.78 ± 0.61	−0.88^b^	.380
Hamilton anxiety rating scale	3.44 ± 3.74	6.26 ± 5.75	−2.70^b^	***.007***
Hamilton depression rating scale	1.73 ± 2.42	5.80 ± 6.15	−3.95^b^	***<.001***
Digital span test‐forward	8.53 ± 1.32	8.00 ± 1.84	−0.91^b^	.362
Digital span test‐backward	6.16 ± 1.74	5.71 ± 2.12	−1.06^b^	.289
Verbal fluency test‐animal	20.22 ± 5.23	19.81 ± 4.75	0.40^c^	.689
Verbal fluency test‐vegetable	15.18 ± 4.04	14.93 ± 3.88	−0.10^b^	.921
Stroop‐spot	13.46 ± 3.07	13.66 ± 3.16	−1.71^b^	.087
Stroop‐word	16.13 ± 4.19	16.36 ± 4.62	−0.20^b^	.843
Stroop‐color word	25.50 ± 7.71	26.74 ± 9.66	−0.33^b^	.741

*Note*: mean ± standard deviations; alcohol (risky/moderate): alcohol consumption level, moderate drinking = less than 20 g/day, risky drinking = 20–60 g/day; smoking (frequent/no): current smoking status, no ≤1 cigarette puff per day, frequent ≥1 cigarette puff per day; mean headache severity refers to the mean headache intensity over the last year.

Abbreviation: Na, not applicable/not available.

^a^ Chi‐squared test.

^b^ Mann–Whitney *U* test.

^c^ Two‐sample *t*‐test.

^d^ Covariate analysis of variance.

#### 3.1.2 Delay discounting task

**FIGURE 1:**
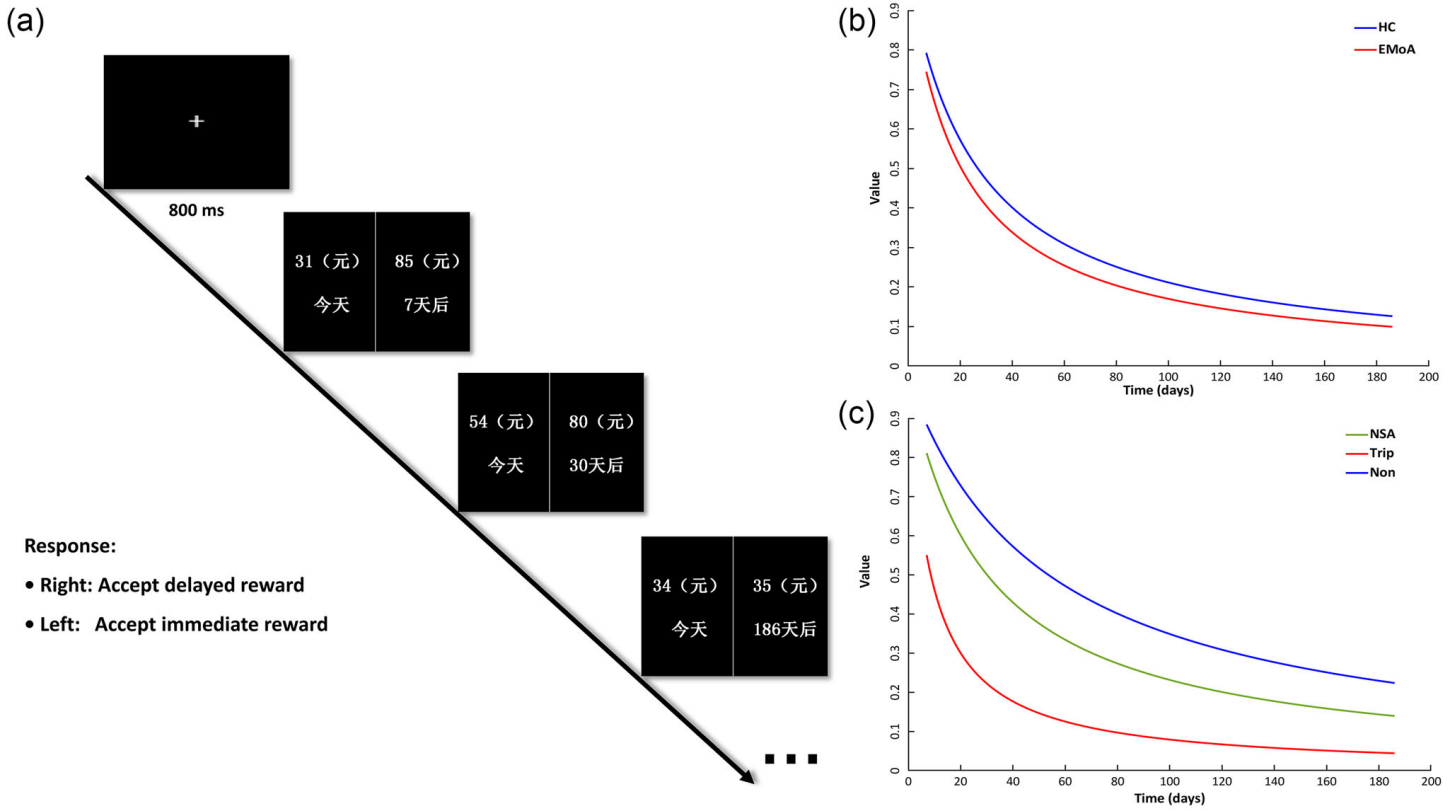
Trial sequence and the results of delay discounting task (DDT). (a) The first screen (cross) indicates that an offer is about to be made. The second‐forth screen presents the actual offers and asks participants choose between them by clicking either the left or right button of the mouse (second screen: ¥ 31‐yuan now vs. ¥ 85‐yuan with 7 days delay; third screen: ¥ 54‐yuan now vs. ¥ 80‐yuan with 30 days delay; fourth screen: ¥ 34‐yuan now vs. ¥ 35‐yuan with 186 days delay). (b) Hyperbolic fitting of delay discount paradigm in episodic migraine without aura (EMoA) patients and healthy control (HC). The temporal discount rate is significantly higher in patients with EMoA. (c) Hyperbolic fitting of delay discount paradigm in EMoA‐NSA, EMoA‐Trip, and EMoA‐non patients. NSA = EMoA patients who received nonsteroidal anti‐inflammatory drugs (EMoA‐NSA); Trip = EMoA patients received triptans; Non = EMoA patients received unused drugs.

For the DDT, the log‐transformed subjective discount rates were significantly higher in patients with EMoA (Table 1, Figure 1), suggesting increased impatience or impulsive choice behavior in this group.

#### 3.1.3 Neuropsychological assessment

The EMoA group was significantly more anxious and depression than the HC group (*p* = .007/*p* < .001). No differences were observed between EMoAs and HCs in the MMSE, DST‐F/B, SCWT‐pot/word/color word, or VFT‐A/V scores (Table 1) (all *p* > .05).

### 3.2 Seed‐based RSFC analysis in the EMoA

**FIGURE 2:**
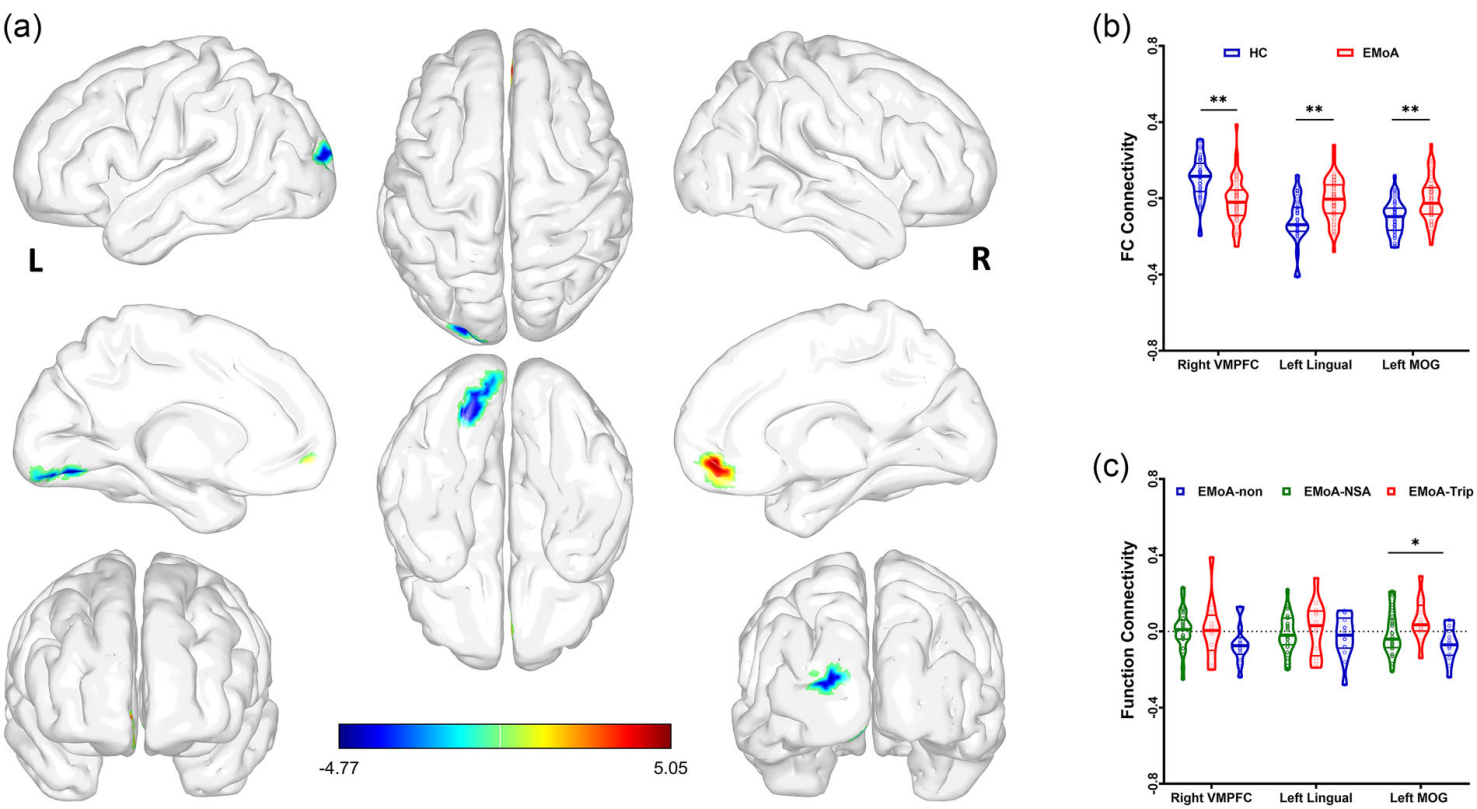
Resting‐state functional connectivity between left ventral striatum (vSTR) and whole brain: (a) The resting‐state functional connectivity (RSFC) between the left vSTR and right ventromedial prefrontal cortex (VMPFC) (BA 11), left lingual gyrus (BA 18), and left middle occipital gyrus (MOG) (BA 18) is changed; (b) reduced positive (VMPFC)/negative (left lingual gyrus and left MOG) functional connectivity strengthen is found in episodic migraine without aura (EMoA) patients relative to healthy controls (HC); and (c) the RSFC between left vSTR and left MOG is reversed in triptans group. NSA = EMoA patients who received nonsteroidal anti‐inflammatory drugs (EMoA‐NSA); Trip = EMoA patients received triptans; Non = EMoA patients received unused drugs. ^*^ *p* < .05; ^**^ *p* < .01.

RSFC analyses were performed for the seed‐whole brain, points located in the left and right vSTR. Compared to HCs, the RSFC between the left vSTR and right middle orbital frontal gyrus (BA 11), left lingual gyrus (BA 18), and left middle occipital gyrus (MOG) (BA 18) was increased in the EMoA, but not in right vSTR (Table 2, Figure 2).

**TABLE 2:** Clusters with significantly changed functional connectivity with the left ventral striatum.

	*x*	*y*	*z*	Voxel	HC	EMoA	*t*	*p*
Right middle orbital frontal gyrus (BA 11)	12	51	−15	67	0.11 ± 0.11	−0.01 ± 0.12	4.97	*<.001*
Left lingual gyrus (BA 18)	−12	−84	−12	59	−0.12 ± 0.12	−0.01 ± 0.11	−4.72	*<.001*
Left middle occipital gyrus (BA 18)	−21	−99	15	42	−0.1 ± 0.09	−0.01 ± 0.11	−4.34	*<.001*

*Note*: mean ± standard deviations.

Abbreviations: EMoA, episodic migraine without aura patients; HC, healthy control.

### 3.3 Correlations between lg‐*k* and clinical characteristics in EMoA patients

The association between the log‐transformed subjective discount rate and clinical characteristics was examined using Spearman's correlation. The log‐transformed subjective discount rate was positively associated with history of migraine (*r*′ = .742, *p* < .001), but not other headache parameters, that is, headache frequency, headache duration, acute pain medication, SF‐MPQ, PRI‐S, PRI‐A, mean headache severity, headache intensity on scan day, HIT, and neuropsychological assessment (Table S1).

### 3.4 Correlations between lg‐*k* and RSFC in EMoA patients

The log‐transformed subjective discount rate was related by the RSFC between the left vSTR and left middle occipital gyri (*r* = .380, *p* = .006). The RSFC between the left vSTR and the left MOG was also positively correlated with a history of migraine (*r′* = .294, *p* = .036) (Table S1).

### 3.5 Subgroups analysis in EMoA with different drug treatments

Thirty‐seven patients received medication at migraine onset: 25 patients received nonsteroidal anti‐inflammatory drugs (EMoA‐NSA), 12 received rizatriptans (EMoA‐Trip), and 14 received unused drugs (EMoA‐Non). There were no differences in headache frequency, headache duration, SF‐MPQ, PRI‐S, PRI‐A, headache intensity on scan day, HIT, or neuropsychological assessment. However, mean headache severity in the last year was lower among patients who used no medication than in other patients. Furthermore, patients who received triptans had deeper subject delay discounting, and the RSFC between the left vSTR and the left MOG was significantly stronger compared than in those who received nonsteroidal anti‐inflammatory drugs or used no medications (Table 3, Figure 2).

**TABLE 3:** Demographics, neuropsychological assessment, and delay discounting task (DDT) performance of three episodic migraine without aura (EMoA) groups.

	EMoA‐NSA	EMoA‐Trip	EMoA‐Non	F/Z	*p*
Sex (F/M)	17/8	10/2	11/3	1.17^a^	.557
Age (years)	33.24 ± 10.87	32.75 ± 11.62	26.64 ± 4.73	4.48^b^	.106
Education (years)	11.36 ± 3.45	12.92 ± 3.34	13.50 ± 2.85	3.88^b^	.144
Alcohol (frequent/no)	1/24	0/12	1/13	0.88^a^	.645
Smoking (frequent/no)	1/24	1/11	1/13	0.33^a^	.848
Family historical (yes/no)	14/11	8/4	7/7	0.75^a^	.688
Total time of migraine pain (years)	6.22 ± 5.25	12.46 ± 10.13	5.93 ± 3.88	5.99^b^	.051
Frequency acute pain (days/month)	5.12 ± 3.07	4.46 ± 3.16	3.92 ± 1.89	0.85^c^	.436
Headache frequency (per month)	5.00 ± 2.89	4.67 ± 3.70	4.64 ± 2.59	0.79^b^	.674
Duration of headache (hours)	25.44 ± 11.13	29.00 ± 19.46	22.00 ± 5.82	0.45^b^	.797
McGill pain questionnaire	11.96 ± 5.05	11.58 ± 6.42	8.85 ± 3.88	1.75^c^	.185
Pain rating index sensory Index	7.04 ± 2.99	6.67 ± 3.68	5.84 ± 2.73	0.67^c^	.515
Pain rating index affective Index	4.92 ± 2.69	4.92 ± 3.55	3.01 ± 2.29	5.21^b^	.074
Mean headache severity (0–10)	6.20 ± 1.32	6.08 ± 1.98	4.91 ± 1.48	7.15^b^	***.028***
Headache intensity on scan day (0–10)	2.64 ± 0.86	2.75 ± 1.42	2.11 ± 0.49	4.09^b^	.129
Headache impact test	60.56 ± 7.41	60.75 ± 9.19	56.48 ± 8.99	2.44^b^	.295
Lg (k)	−2.18 ± 0.86	−1.17 ± 0.63	−2.12 ± 0.79	12.26^b^	***.002***
Mini‐mental state Examination	29.72 ± 0.68	29.67 ± 0.78	30.00 ± 0.00	2.53^b^	.283
Hamilton anxiety rating scale	6.28 ± 5.86	5.42 ± 6.47	6.95 ± 5.19	1.61^b^	.446
Hamilton depression rating scale	6.57 ± 6.33	4.42 ± 5.57	5.62 ± 6.50	1.60^b^	.450
Digital span test‐forward	8.20 ± 1.66	7.83 ± 1.70	7.79 ± 2.33	0.51^b^	.775
Digital span test‐backward	5.68 ± 2.10	5.25 ± 1.91	6.14 ± 2.38	2.01^b^	.367
Verbal fluency test‐animal	19.36 ± 5.22	19.00 ± 4.16	21.32 ± 4.29	2.51^b^	.285
Verbal fluency test‐vegetable	15.00 ± 4.10	13.50 ± 3.29	16.04 ± 3.78	1.41^c^	.253
Stroop‐spot	14.00 ± 2.16	12.41 ± 4.24	14.10 ± 3.56	2.00^b^	.369
Stroop‐word	17.36 ± 5.73	16.05 ± 3.18	14.83 ± 2.90	1.40^c^	.257
Stroop‐color word	27.89 ± 9.70	28.92 ± 12.43	22.82 ± 5.55	2.39^b^	.303

*Note*: Mean ± standard deviations; alcohol (frequent/no): alcohol consumption level, no = less than once per week, frequent = more than once per week; mean headache severity refers to the mean headache intensity over the last year. EMoA‐NSA: episodic migraine without aura patients who patients received nonsteroidal anti‐inflammatory drugs (EMoA‐NSAIDs), 12 patients receive triptans (EMoA‐Triptans), and 14 patients receive unused drugs (EMoA‐Non).

^a^ Chi‐squared test.

^b^ Mann–Whitney *U* test.

^c^ ANOVA.

### 3.6 Mediation analysis

Three‐step method examining the mediating effect of RSFC between medication history and log‐transformed subjective discount rate revealed that the RSFC has a mediating effect (−0.021, 95% confidence interval [−0.049, −0.000]) between medication history and log‐transformed subjective discount rate (Table 4).

**TABLE 4:** The mediating effect of resting‐state functional connectivity (RSFC) between medication history and Lg‐*k*.

				95% CI			
Item	Symbols	Meaning	Effect size	Low	High	*z*/*t*	*p*
Medication history ≥ RSFC ≥ Lg‐*k*	a*b	Indirect effects	−0.021	−.20	.00	−0.41	.682
Medication history ≥ RSFC	*a*	X ≥ M	−0.009	−.02	−.00	−2.02	*.048*
RSFCL ≥ Lg‐*k*	*b*	M ≥ Y	2.359	.18	4.54	2.12	*.039*
Medication history ≥ Lg‐*k*	*c*′	Direct effect	0.001	−.07	.07	0.02	.988
Medication history ≥ Lg‐*k*	*C*	Total effect	−0.021	−.09	.05	−0.58	.566

*Note*: RSFC: function connectivity between left vSTR and left middle occipital gyrus, Lg‐*k*: log‐transformed subjective discount rate (*k* value).

Abbreviation: CI, confidence interval.

## 4 DISCUSSION

This study assessed whether impulsive decision‐making behavior and associated brain mechanisms were altered in patients with EMoA. We first showed that patients with EMoA had an elevated subjective discount rate, which was positively related to a history of migraine, but there was no difference in neuropsychological assessments. We further examined whether there were any changes in the RSFC of EMoA. In patients with EMoA, the RSFC in the left vSTR, ventromedial prefrontal cortex (VMPFC), and left middle occipital regions was reduced. The RSFC between the left vSTR and the left occipital region was also related to the subjective discount rate and history of migraine. We further showed that the subjective discount rate and RSFC between the left vSTR and the left middle occipital lobe in EMoA patients could be modified by triptans. Triptan may increase the discount rates in patients with EMoA by reversing the RSFC between the left vSTR and left occipital lobe.

These findings revealed that the delay‐discounting in patients with EMoA was significantly higher than that in healthy participants. In the delay‐discounting task, a larger subjective discount rate indicates steeper delay discounting and is frequently interpreted as reflecting an impulsive preference for immediate rewards over delayed gratification. Previous studies revealed that patients with medication overuse, headaches, or chronic migraines showed steeper delayed discounting behavior than HCs (Amlung et al., 2019; Bottiroli et al., 2023; Lempert et al., 2019; Potvin et al., 2015; Zeng et al., 2022). In the present study, we determined the impulsive decision behavior performance in patients with migraine without aura using the DDT, which supplements the previous finding of the subjective discount rate in patients with migraine without aura (Chen et al., 2023). Moreover, we found an association between the preference for a higher delay discounting rate and a longer migraine history, but not with any of the neuropsychological tests. In other words, the increased subjective discount rate observed in migraineurs compared to controls was directly related to the duration of migraine. These results are consistent with previous reports that steep discounting is not simply an indicator of poor cognitive functioning (Garzon et al., 2023; Yeh et al., 2021). This indicates that the higher rate of delayed discounting may be an intrinsic, stable behavioral feature in patients with migraine rather than restricted to migraine patients with drug abuse, who may be affected by migraine pathology rather than cognitive function. Future studies with larger samples are needed to clarify the relationship between delayed discounting rates and different types of migraine.

Abnormal time perception may be the neuropsychological mechanism underlying increased delayed discounting rates in patients with migraine. Subjective time perception refers to how long a duration feels subjectively to an individual, which can differ from objective time as measured by a clock (Paasche et al., 2019). A growing number of studies indicate that time perception, future‐oriented cognitive processes, and tolerance of uncertainty‐delayed rewards may serve as important psychological mechanisms of delay discounting (Amlung et al., 2019; Haynes et al., 2023; Paasche et al., 2019). Delay discounting may be affected by how one perceives time. When subjective time passes quickly, individuals tend to overestimate the duration of an objective interval (Paasche et al., 2019). This can lead to a lack of patience with uncertain delayed rewards and a tendency to seek immediate short‐term rewards (Zeng et al., 2022). Patients with migraines have impaired time perception, which could result in longer duration estimates and slower passage of time, which may be the psychological mechanism for the increased delay discounting rate (Zhang et al., 2012). Similarly, abnormal perception of time is significantly associated with an increased rate of delayed discounting in patients (Zeng et al., 2022). Further clarification of the psychological mechanisms underlying the increased rate of delayed discounting in patients with migraines may require simultaneous exploration of more behavioral paradigms, such as the temporal reproduction task.

Dysfunctional interconnections between the VMPFC and striatum may be responsible for impulsive choices in migraines (Wang et al., 2020). The striatum and VMPFC are important components of the reward network (Guizar Rosales et al., 2022; Hare et al., 2009; Hiser & Koenigs, 2018; Ikink et al., 2019; Niddam et al., 2023; Wang et al., 2020). The striatum is crucial for reward experiences and learning, and the VMPFC is preferentially involved in value computation processes, such as delayed reward valuation, decision value encoding, and choice preference processes (Hare et al., 2009; Hiser & Koenigs, 2018; Ikink et al., 2019; Schneider & Koenigs, 2017). Functional interconnections between the VMPFC and striatum are responsible for impaired reward value updating and adaptive learning (Qiu & Wang, 2021). The striatum and VMPFC cooperate to participate in the delayed decision‐making process, particularly in value assessments (Hiser & Koenigs, 2018). The reduction/disruption of functional connectivity is closely related to an increase in delay discounting and predicts the rate of delay discounting in subjects (Guizar Rosales et al., 2022; Qiu & Wang, 2021). Our results are consistent with previous reports suggesting that reduced VMPFC and striatal activity are correlated with higher delay discounting in substance detection and gambling addiction (Luijten et al., 2017). The weaker functional connectivities between the striatum and VMPFC have been observed in migraine groups (Lee et al., 2023; Schneider & Koenigs, 2017). Hence, we propose that the reduced strength of functional connectivity in the striatum and VMPFC in patients with migraines may lead to a reduction in individuals’ expectation of long‐term value, which in turn leads to an increase in delayed discounting. In addition, this abnormality in functional connectivity may be responsible for its overuse in patients with chronic migraine (Niddam et al., 2023).

The activation of occipital lobe function and the abnormality of the functional connection between the occipital lobe and striatum may be closely related to increase delayed discounting in patients. Prior studies indicate that the occipital cortex is involved in the evaluation of long‐term harvest, and increased activity of the occipital cortex is beneficial for the high expectation of long‐term harvest, especially monetary cues (Qiu & Wang, 2021). The present results demonstrate that the intensity of the negative functional connection between the vSTR and occipital lobe was significantly lower in the headache than the healthy group and was strongly associated with an increase in patient delay discounting. This result emphasizes the importance of attentional processing of visual information and sensory neurocircuits for nondrug cues with reward properties in patients with migraines. Resting state functional magnetic resonance studies found that activity in the occipital lobe was significantly impaired in the patient group, and the activity intensity in the occipital lobe decreased significantly when patients processed visual stimulus information (Hu et al., 2023; Lee et al., 2023). These results may elucidate the pathology of gain and loss processing in patients with migraine. However, for patients with migraine, further studies, such as task‐state MRI or electroencephalography, are warranted to further explore the potential relationship between changes in functional connectivity between the striatum and occipital lobe and delayed discounting and its predictive effect on the conversion to substance‐dependent chronic migraine.

The increased delayed discounting rate in patients with migraines may be caused by dysfunction of the 5‐hydroxytryptamine (5‐HT) system. Fibers of 5‐HT‐ergic neurons are distributed in the hypothalamus and limbic system of the central and play an important role in impulsive decision/delay discounting (Bacque‐Cazenave et al., 2020; Pommer et al., 2021; Salvan et al., 2023). Previous studies on decision‐making indicate that the activation of the 5‐HT system can increase the expectation of future benefits and make more patient choices (Desrochers et al., 2021; Miyazaki et al., 2011). 5‐HT blood levels in patients with migraine are significantly lower than those in normal controls, and the decrease is more obvious during the attack period than during the interval period (Puledda et al., 2023; Schramm et al., 2023). Therefore, it can be hypothesized that the reduction of 5‐HT content in the brain of migraine patients may also make them more prone to instant rewards and more prone to making impulsive decisions, with a reduced proportion of delayed choices. Interestingly, our results indicated that patients with migraines who used triptans for pain relief had significantly high rates of delayed discounting than those who did not. Triptans mainly activate serotonin receptors in the brain stem and limbic system and activate serotonin neurons to relieve headaches and are the most used drugs in the acute phase of migraine (Puledda et al., 2023). Hence, we speculate that triptans can alleviate pain by activating 5‐HT neurons, whereas frequent use of triptans may decrease serotonin receptor function, thereby decreasing patients’ expectations of long‐term value and increasing the delay discount rates or behavioral impulsivity. Additionally, our results demonstrate that triptans appear to be increasing the delayed discounting in patients with migraine. This could be achieved by reversing the effect of paroxysmal migraine on the strength of the functional connection between the striatum and the occipital lobe; therefore, increasing the impulsivity of behavior. This finding is consistent with previous research showing that drug use may increase the impulsive behavior in patients (Pommer et al., 2021). This provides additional behavioral evidence for the choice of medication in the acute phase of migraine; therefore, the effects of medicine use in the acute phase on the incidence of chronic headache and drug abuse should be further explored.

Despite its strengths, this study also has the following disadvantages: First, the sample size was small, especially for patients who used triptan drugs alone, mainly because some patients had difficulty adhering to long‐term triptan drug use for personal reasons; therefore, larger samples may be needed in the future; second, long‐term follow‐up was not available to explore whether an increase in the delay discount predicted the transition from episodic migraine to chronic and drug‐dependent headaches. Third, task‐state functional magnetic resonance studies may be required to further explore the neural mechanisms underlying delayed discount increases in patients with migraines.

## 5 CONCLUSIONS

This study is the first to show a significant increase in the rate of delayed discounting in patients with EMoA and a significant correlation with the duration of the disease. Additionally, multimodal magnetic resonance analysis revealed that changes in the functional connections between the striatum and the ventromedial prefrontal and occipital lobes were associated with an increase in the delay discounting rate. It is notable that triptan use in the acute phase of migraine attacks induces an increase in delayed discharges and abnormal changes in the functional connection between the striatum and the occipital lobe in EMoA patients. This study further expanded the behavioral characteristics of patients with EMoA; that is, an increase in delayed discounting rate also exists in patients with EMoA, and the increase in delayed discounting rate may predict the occurrence of chronic migraine and drug abuse in the future. These findings provide a new perspective on the effects of triptans; however, a larger sample size and longer term follow‐up are required in future studies.

## AUTHOR CONTRIBUTIONS

**Lu Wang**: Methodology; investigation; writing—original draft; conceptualization. **Chenyang Dai**: Data curation. **Manman Gao**: Data curation. **Zhi Geng**: Data curation. **Panpan Hu**: Investigation; funding acquisition. **Xingqi Wu**: Funding acquisition; investigation; writing—review and editing; data curation; formal analysis; conceptualization. **Kai Wang**: Funding acquisition; investigation; writing—review and editing.

## CONFLICT OF INTEREST STATEMENT

The authors declare that they have no conflicts of interest.

### PEER REVIEW

The peer review history for this article is available at https://publons.com/publon/10.1002/brb3.3367.

## Supporting information

Table S1 The correlation between lg‐*k* and demographics, neuropsychological assessment, and RSFC in EWoA groups.
Table S2 Clusters with significantly changed functional connectivity with the left vSTR in three EWoA groups.
Table S3 The mediating effect of RSFC between medication history and lg‐*k*.

## Data Availability

The data supporting the findings of this trial are available upon request from the corresponding authors.
