# Supplementary material for: Patients with episodic migraine without aura have an increased rate of delayed discounting
Source: Brain Behav. 2024 Jan 2;14(1):e3367. doi: 10.1002/brb3.3367 (PMC10761331; doi:10.1002/brb3.3367)
Supplement: Supplementary file 1 — Table S1 The correlation between lg‐k and demographics, neuropsychological assessment, and RSFC in EWoA groups. Table S2 Clusters with significantly changed functional connectivity with the left vSTR in three EWoA groups. Table S3 The mediating effect of RSFC between medication history and lg‐k. [file BRB3-14-e3367-s001.docx]

Patients with episodic migraine without aura have an increased rate of delayed discounting

**Supplemental material**

Lu Wang Ph.D. ^a,d,e,△^,Chenyang Dai M.M. ^a,d,e, △^, Manman Gao M.M. ^a,d^, Zhi Geng M.M. ^a,d^, Pan Wu M.M. ^a,d^, Panpan Hu Ph.D. ^a,d,e^, Ling Wei Ph.D. ^a,d,e,*^,Xingqi Wu M.D. ^a,d,e,*^, Kai Wang M.D. , Ph.D. ^a,b,c,d,e,f,*^

^a^ Department of Neurology, The First Affiliated Hospital of Anhui Medical University, Hefei 230022, China

^b^ School of Mental Health and Psychological Sciences, Anhui Medical University, Hefei 230000, China

^c^ Institute of Artificial Intelligence, Hefei Comprehensive National Science Center, Hefei, China. 230088

^d^ Anhui Province Key Laboratory of Cognition and Neuropsychiatric Disorders, Hefei 230022, China

^e^ Collaborative Innovation Center of Neuropsychiatric Disorders and Mental Health, Hefei 230022, China

^f^ Anhui Provincial Institute of Translational Medicine, Anhui Medical University, Hefei, 230032, China

^△^ These authors contributed to the work equally, and should be regarded as co-first authors.

***Correspondence to:**

Kai Wang, Ph.D., M.D. E-mail: [wangkai1964@126.com](mailto:wangkai1964@126.com)

Xingqi Wu, M.D. E-mail: [wuxingqi09@163.com](mailto:wuxingqi09@163.com)

Ling Wei, Ph.D.  E-mail: [ahykdxwl@126.com](mailto:ahykdxwl@126.com)

Tel/Fax: +86-0551-62923704

Department of Neurology, The First Affiliated Hospital of Anhui Medical University, Jixi Road. No 218, Hefei 230000, China

***Number of tables: 3**

| **sTable 1: the correlation between Lg K and Demographics, Neuropsychological assessment, and RSFC in EWoA groups** | | | |
| --- | --- | --- | --- |
|  | r | p | n |
| Age (years) | -0.10 | 0.503 | 51 |
| Education (years) | 0.09 | 0.528 | 51 |
| Total time of migraine pain (years) | 0.74 | ***<0.001*** | 51 |
| Frequency Acute pain (days/month) | -0.11 | 0.453 | 51 |
| Headache frequency (per month) | -0.23 | 0.105 | 51 |
| Duration of headache (hours) | 0.06 | 0.681 | 51 |
| McGill pain questionnaire | 0.08 | 0.557 | 51 |
| Pain Rating Index Sensory Index | 0.07 | 0.639 | 51 |
| Pain Rating Index Affective Index | 0.10 | 0.466 | 51 |
| Mean headache severity (0–10) | 0.05 | 0.704 | 51 |
| Headache intensity on scan day (0–10) | 0.08 | 0.557 | 51 |
| Headache Impact Test | 0.22 | 0.123 | 51 |
| Mini-Mental State Examination | 0.12 | 0.403 | 51 |
| Hamilton Anxiety Rating Scale | 0.08 | 0.566 | 51 |
| Hamilton Anxiety Rating Scale | 0.04 | 0.796 | 51 |
| Digital Span Test-Forward | 0.08 | 0.568 | 51 |
| Digital Span Test-Backward | 0.05 | 0.712 | 51 |
| Verbal Fluency Test-Animal | 0.14 | 0.316 | 51 |
| Verbal Fluency Test-Vegetable | -0.07 | 0.645 | 51 |
| Stroop-spot | -0.16 | 0.262 | 51 |
| Stroop-word | -0.12 | 0.403 | 51 |
| Stroop-Color Word | -0.21 | 0.148 | 51 |
| Right middle orbital frontal gyrus (BA 11) | 0.03 | 0.819 | 51 |
| Left lingual gyrus (BA 18) | 0.10 | 0.484 | 51 |
| Left middle occipital gyrus (BA 18) | 0.38 | ***0.006*** | 51 |
| Note: spearman correlation analysis | | | |
| Abbreviation: DDT: Delay Discounting Task, RSFC, function connectivity between left vSTR and left middle occipital gyrus; Lg K, log-transformed subjective discount rate (K value) | | | |

| **sTable 2: Clusters with significantly changed functional connectivity with the left ventral striatum in three EWoA groups** | | | | | |
| --- | --- | --- | --- | --- | --- |
|  | EWoA-NSA | EWoA -Trip | EWoA-Non | F | p |
| Right middle orbital frontal gyrus (BA 11) | 0.00±0.11 | 0.01±0.16 | -0.07±0.10 | 4.72 | 0.095 |
| Left lingual gyrus (BA 18) | -0.01±0.1 | 0.02±0.14 | -0.03±0.11 | 0.53 | 0.590 |
| Left middle occipital gyrus (BA 18) | -0.01±0.11 | 0.06±0.11 | -0.07±0.09 | 4.26 | ***0.020*** |
| Note: mean ± standard deviations; EWoA: episodic migraine without aura patients, HC: Healthy Control | | | | | |

| **sTable 3: The mediating effect of RSFC between medication history and Lg k** | | | | | | | | | | | | | | | |
| --- | --- | --- | --- | --- | --- | --- | --- | --- | --- | --- | --- | --- | --- | --- | --- |
|  | Lg K | | | | | RSFC | | | | | Lg K | | | | |
|  | *B* | SE | *t* | *p* | *β* | *B* | SE | *t* | *p* | *β* | *B* | SE | *t* | *p* | *β* |
| Count | -1.85 | 0.18 | -10.54 | 0.000 | Na | 0.02 | 0.02 | 1.00 | 0.324 | na | -1.90 | 0.17 | -11.09 | 0.000 | na |
| Medication History | -0.02 | 0.04 | -0.58 | 0.566 | -0.082 | -0.01 | 0.00 | -2.02 | 0.048 | -0.28 | 0.00 | 0.04 | 0.02 | 0.988 | 0.002 |
| RSFC |  |  |  |  |  |  |  |  |  |  | 2.36 | 1.11 | 2.12 | 0.039 | 0.304 |
| *R*^2^ | 0.007 | | | | | 0.077 | | | | | 0.092 | | | | |
| Adjust *R*^2^ | -0.014 | | | | | 0.058 | | | | | 0.054 | | | | |
| *F* | *F* _(1,49)_ =0.33, *p*=0.566 | | | | | *F* _(1,49)_ =4.10, *p*=0.048 | | | | | *F* _(2,48)_ =2.426, *p*=0.099 | | | | |
| Note: RSFC, function connectivity between left vSTR and left middle occipital gyrus; Lg K, log-transformed subjective discount rate (K value) | | | | | | | | | | | | | | | |
